# The relative burden of community-acquired pneumonia hospitalizations in older adults: a retrospective observational study in the United States

**DOI:** 10.1186/s12877-018-0787-2

**Published:** 2018-04-16

**Authors:** Joshua D. Brown, James Harnett, Richard Chambers, Reiko Sato

**Affiliations:** 1 Department of Pharmaceutical Outcomes & Policy, University of Florida College of Pharmacy, Gainesville, USA; 2 Global Health and Value, Real World Data and Analytics, Pfizer Inc., New York, NY USA; 3 Global Product Development, Statistical Research Data Center, Pfizer, Inc., Collegeville, PA USA; 4 Global Health and Value, Outcomes and Evidence, Pfizer, Inc., Collegeville, PA USA

**Keywords:** Community-acquired pneumonia, Burden of illness, Geriatrics, Vaccinations

## Abstract

**Background:**

The risk of community-acquired pneumonia (CAP) increases with age and significantly impacts morbidity and mortality in the elderly population. The burden of illness and cost of preventing CAP has not been compared to other serious diseases.

**Methods:**

This retrospective analysis used claims data from 2014 to 2015 and compared hospitalizations for CAP, myocardial infarction (MI), stroke, and osteoporotic fractures (OF) in adults aged ≥65 years enrolled in a Medicare Advantage insurance plan. Individuals who had not already been hospitalized for one of these conditions and did not have evidence of long-term care were included in the study. Hospitalizations for each condition were described by length of stay, readmissions, mortality, and total costs. Preventive measures included vaccinations for CAP and medications for MI, stroke, and OF.

**Results:**

A total of 1,949,352 individuals were included in the cohort. In 2015, the rate of CAP-related hospitalizations was the highest at 846.7 per 100,000 person-years compared to 405 for MI, 278.9 for stroke, and 343.9 for OF. Vaccination costs for CAP were $40.2 million including $14.1 million for pneumococcal and $26.1 million for influenza vaccines. The cost of preventive medications for MI and stroke reached over $661 million and OF totaled $169 million.

**Conclusions:**

Although CAP has a higher burden of hospitalization and total costs than MI, stroke, and OF in the elderly population, prevention efforts were disproportionately smaller for CAP. Prioritization of CAP prevention is needed to substantially reduce the burden of CAP.

## Background

An estimated 1.3 million annual cases of community-acquired pneumonia (CAP) occur among adults aged 65 years and older, contributing significant morbidity, mortality and economic burden in the United States [1]. Nearly 40% of CAP episodes in seniors will result in a hospitalization with an average length of stay of 5.6 days [2], incurring medical costs in excess of $18,000 per inpatient episode [1, 3]. The aggregate economic burden of CAP in the Medicare fee-for-service population alone is estimated to be over $13 billion annually and is expected to grow with the aging population [1, 4].

CAP includes infections not associated with encounters in the healthcare system (i.e., nosocomial or iatrogenic infections) for community-dwelling individuals. CAP risk increases with age from 18.2 cases per 1000 person-years among those aged 65–69 years up to 52.3 cases per 1000 person-years among those 85 years and older [3]. Additional risk factors include chronic obstructive pulmonary disease (COPD), asthma, diabetes, congestive heart failure (CHF), immunosuppression, cancer, and smoking [4–6]. These risk factors tend to be highly prevalent in the older population and increase the risk of more severe disease and death due to CAP [1, 3, 7].

Other serious medical events that frequently occur in seniors are myocardial infarction (MI), stroke, and osteoporotic fractures (OF). According to the 2010 National Hospital Discharge Survey, there were an estimated 354,000 MI hospitalizations, 663,000 stroke hospitalizations, and 586,000 facture related hospitalizations in adults aged ≥65 years [8]. These conditions are considered common targets for primary prevention by healthcare providers and managed care organizations because of the high prevalence of disease, modifiable risk factors, availability of proven therapies and strategies to reduce the risk of the medical event, potential for high disability, and substantial economic burden [9, 10]. In comparison, there were 621,000 CAP hospitalizations in the same age group, which is similar or greater than that of MI, stroke, and OF.

In health systems with constrained resources, resource utilization for preventive efforts would ideally be proportional to the disease burden in the population. Prior studies have estimated the burden of illness for CAP [1, 3], but the incidence and cost of hospitalizations due to CAP have not been compared in the context of other serious diseases and how the relative burden of disease compares to the expenditures to prevent those diseases. This study sought to compare the relative burden of hospitalizations for CAP compared to MI, stroke, and OF, as well as to assess the relative expenditures of preventive measures for each disease in a population of older adults in a large, national, Medicare Advantage plan.

## Methods

### Data source

This retrospective cohort study utilized administrative healthcare claims data from Optum’s Clinformatics™ Data Mart (CDM) between 2014 and 2015 in a study population comprised of Medicare Advantage with Prescription Drug Plan (MAPD) beneficiaries from a large national managed care company affiliated with Optum. MAPD plans are alternative plans to “traditional” Medicare insurance coverage, often call “Part C,” where federal payments are provided to private insurance companies. These organizations provide an insurance benefit which includes inpatient, outpatient, prescription coverage, and often additional dental, vision, or other benefits usually within Health Maintenance Organization (HMO) or Preferred Provider Organization (PPO) structures. Enrollment into MAPD is optional and roughly one-third of Medicare beneficiaries enroll in MAPD coverage [11]. Compared to traditional Medicare enrollees, MAPD members tend to be older, more racially diverse, and have lower socioeconomic status [12].

The Optum Clinformatics™ Data Mart is statistically de-identified under the Expert Determination method consistent with HIPAA and managed according to Optum customer data use agreements. These administrative claims submitted for payment by providers and pharmacies are verified, adjudicated, adjusted, and de-identified prior to inclusion. The population is geographically diverse, spanning all 50 states in the U.S.. In addition to medical claims and pharmacy claims, the data include tables with member eligibility and demographic information. The data also include standard pricing for all medical claims, pharmacy claims, and inpatient confinements. The data use an encrypted member identification number to longitudinally track members across years. Information on the month and year of death are appended to the database by Optum prior to de-identification using the Social Security Administration Death Master File.

### Study population

Adults aged 65 to 89 years with continuous MAPD enrollment from January 1, 2014 through December 31, 2015 were included in this study. Censoring due to death in 2015 was permitted since it was an outcome of interest. Hospitalization for CAP, MI, stroke, and OF were identified during the 2015 calendar year. The 2014 calendar year was used to assess cohort clinical characteristics as well as to capture preventive measures for each of the four conditions. Subjects who had a hospitalization for CAP, MI, stroke or OF or a cumulative stay in a skilled nursing facility for ≥90 days during 2014 were excluded to create a cohort that was community-dwelling and free of the hospitalizations of interest at the start of follow-up in 2015.

### Cohort characteristics

Age, gender, region, and low income subsidy status were assessed as of January 1, 2015 from the enrollment data. Individuals were categorized into age groups: 65–69, 70–74, 75–79, 80–84, and 85–89 years; geographic region was based on Census regions (North Central, Northeast, South, West, Unknown). Key comorbidities were identified using ICD-9 and corresponding ICD-10 codes. Deyo-Charlson comorbidities were calculated as a weighted summary score for each individual [13, 14]. Coding algorithms of the comorbidity classifications are summarized in Appendix 1 Table 4.

### Hospitalization metrics

CAP, MI, stroke, and OF hospitalizations were identified beginning on January 1, 2015 in the medical claims using diagnosis codes listed in Appendix 1 Table 4. Only hospital admissions with a primary diagnosis of one of these four conditions were included. Hospitalizations for CAP were identified based on prior work [1], with slight modification to use a primary diagnosis for pneumonia or a primary diagnosis of septicemia or respiratory failure with a diagnosis of pneumonia during the same hospital visit. To distinguish CAP from potential hospital-acquired pneumonia, index CAP hospitalizations with claims indicating other hospitalization, skilled nursing facility, or hospice care in the 14 days preceding a pneumonia hospitalization were excluded.

A 30-day post-discharge period was used to assess hospital readmission for any condition, similar to “all cause readmission” metrics already in use as measures of care quality after a hospitalization [15]. After a 30-day period had elapsed with no readmissions, new hospitalizations for any study condition were counted. All-cause deaths after the index hospitalization were also recorded. However, due to data privacy requirements, dates were restricted at the month level. Thus, we recorded deaths that occurred in the same month or the following month of index admissions of interest.

Each hospitalization and readmission included the length of stay (LOS) and the sum of the amounts paid by the individual and the health plan. Total episode costs included the costs of the index hospitalization and any readmissions observed during the 30-day window. Costs for preventive measures were summarized as costs to the health insurer, member out-of-pocket (OOP) cost, total costs (health insurer plus member OOP), and summarized as per member per year (PMPY) expenditures based on the total population.

### Preventive medication/vaccinations

Preventive measures for CAP included influenza and pneumococcal vaccinations, consistent with Infectious Diseases Society of America and the American Thoracic Society Consensus Guidelines on the Management of Community-Acquired Pneumonia [16]. Preventive medications for MI and stroke comprised of antihypertensives, statins, aspirin, anticoagulants, and antiplatelet medications. OF preventive measures included bisphosphonates, selective estrogen receptor modulators, and injectable therapies (denosumab and teriparatide). All medications were identified by Medi-Span Generic Product Identifier (GPI) codes in the pharmacy claims. Injectable medications and vaccinations were also identified by Current Procedural Terminology codes and Healthcare Common Procedure Coding System codes in the medical claims. Codes for preventive medications are available in Appendix 2 Table 5.

Total summed costs for hospitalizations, readmissions, and total combined costs were described as well as per event costs. Admission, re-admission, and mortality rates were standardized to events per 100,000 life-years to allow direct comparison between conditions within the cohort. Data management and analysis were conducted using SAS Enterprise Guide 5.1 (SAS Institute, Cary, NC). This manuscript was drafted in accordance with STROBE reporting guidelines (http://www.strobe-statement.org/) for observational studies.

## Results

After applying sample selection requirements, 1,949,352 subjects were included in the final study cohort (Appendix 3 Table 6), contributing 1,940,589 person-years after correction for those who died during follow-up in 2015. Baseline demographic and clinical characteristics of the cohort are provided in Table 1. The mean age was 75.9 years, 57.8% of the cohort was female, and the cohort was geographically diverse across regions. The average Deyo-Charlson comorbidity index score was 1.3. Diabetes (25.0%), chronic pulmonary disease (14.7%), coronary artery disease (14.2%), and renal disease (11.3%) were the most prevalent comorbid conditions among those selected for evaluation.

**Table 1:** Cohort demographic and clinical characteristics (*N* = 1,949,352)

	Variable Description	N (%)
Age		
	65–69	402,909 (20.7%)
	70–74	566,324 (29.1%)
	75–79	397,143 (20.4%)
	80–84	288,020 (14.8%)
	85–89	294,956 (15.1%)
Gender		
	Female	1,126,172 (57.8%)
	Male	823,180 (42.2%)
Geographic region		
	North Central	382,856 (19.6%)
	Northeast	286,616 (14.7%)
	South	632,477 (32.5%)
	West	643,917 (33.0%)
	Unknown	3486 (0.2%)
Low Income Subsidy		
	Yes	258,476 (13.3%)
	No	1,577,011 (80.9%)
	Unknown	113,865 (5.8%)
Deyo-Charlson comorbidity index score		
	Mean (SD)	1.3 (1.9)
Comorbidities		
	Myocardial infarction	55,652 (2.9%)
	Peripheral vascular disease	124,624 (6.4%)
	Cerebrovascular disease	175,251 (9.0%)
	Dementia	31,640 (1.6%)
	Rheumatologic disease	49,924 (2.6%)
	Peptic ulcer disease	17,117 (0.9%)
	Hemiplegia or paraplegia	6503 (0.3%)
	Renal disease	219,910 (11.3%)
	Chronic pulmonary disease	285,668 (14.7%)
	Asthma	38,117 (2.0%)
	Heart Failure	120,061 (6.2%)
	Cardiomyopathy	44,327 (2.3%)
	Diabetes	486,478 (25.0%)
	Diabetes with chronic complications	150,350 (7.7%)
	Liver Disease	55,746 (2.9%)
	Alcoholism	11,983 (0.6%)
	Coronary artery disease	277,519 (14.2%)
Immunocompromising conditions, N(%)		
	Asplenia	170 (< 0.1%)
	Sickle cell disease	192 (< 0.1%)
	HIV/AIDS	1951 (0.1%)
	Cancer	131,009 (6.7%)
	Leukemia, leukemia or myeloma	20,473 (1.1%)
	Advanced stage renal disease	4908 (0.3%)
	Immunosuppressive medication use	58,127 (3.0%)
Death in 2015		16,125 (0.8%)
Total person years of follow-up		1,940,589 years

Abbreviations; *SD*: standard deviation

There were 15,701 older adults who experienced a CAP hospitalization, with a total of 16,430 CAP hospitalizations during 2015 – a rate of 846.7 hospitalizations per 100,000 person-years (Table 2). Comparatively, there were 7859 hospitalizations for MI (405 per 100,000 person-years), 5412 hospitalizations for stroke (278.9 per 100,000 person-years), and 6674 hospitalizations for OF (343.9 per 100,000 person-years). The mean [SD] LOS for CAP hospitalizations was 5.2 [6.2] days and statistically longer than MI (4.5 [6.3] days, *p* < 0.001), stroke (4.1 [5.6] days, p < 0.001), and OF (4.2 [3.4] days, *p* = 0.002). The 30-day readmission rate percent was 10.0% for CAP, 10.5% for MI, 7.7% for stroke, and 7.9% for OF. The mortality rate per 100,000 persons for CAP was significantly higher than all comparison diseases: 22.5 for CAP versus 5.3 for MI, 9.7 for stroke, and 5.8 for OF.

**Table 2:** Comparison of CAP, MI, stroke, and OF related hospitalizations in 2015

Characteristic	CAP	MI	Stroke	OF
Persons hospitalized	15,701	7683	5317	6614
Number of hospitalizations	16,430	7859	5412	6674
Incidence of hospitalization rate per 100,000 person-years	846.7	405.0	278.9	343.9
Number of readmissions	1874	950	458	574
30-day readmission rate (%)	10.0	10.5	7.7	7.9
Number of deaths within 30 days of discharge	439	104	189	112
Mortality rate during same or following month per 100,000 persons	22.5	5.3	9.7	5.8
Length of stay per hospitalization				
Mean (SD)	5.2 (6.2)	4.5 (6.3)	4.1 (5.6)	4.2 (3.4)
Median	4	3	3	4
Length of stay per readmission				
Mean (SD)	4.9 (4.3)	4.2 (4.1)	4.2 (4.5)	4.7 (4.1)
Median	4	3	3	4
Cost per hospitalization				
Mean (SD)	13,825 (17,341)	26,114 (28,118)	15,138 (15,810)	19,156 (14,845)
Cost per readmission				
Mean (SD)	12,422 (13,260)	12,177 (14,925)	11,681 (11,525)	13,446 (13,301)
Cost per episode				
Mean (SD)	15,241 (19,078)	27,586 (29,423)	16,126 (17,225)	20,312 (16,384)
Sum of hospitalization costs ($)	227,145,454	205,230,868	81,926,896	127,847,139
Sum of readmission costs ($)	23,280,008	11,568,507	5,350,202	7,718,322
Total costs ($)	250,425,463	216,799,375	87,277,098	135,565,461

List of abbreviations*: CAP*: community-acquired pneumonia; *MI*: myocardial infarction; *OF*: osteoporotic fracture; *SD*: standard deviation

**Fig. 1:**
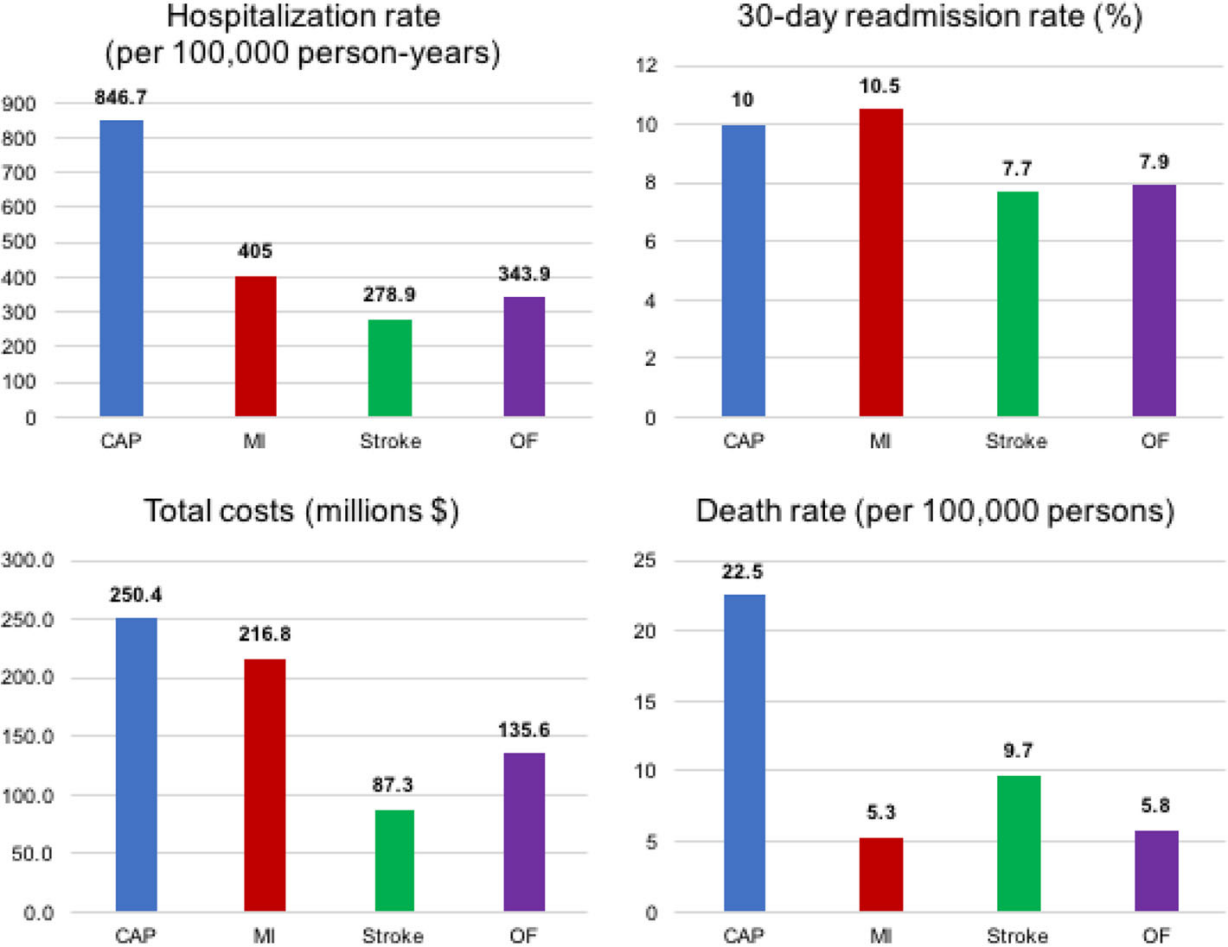
Comparison of hospitalization metrics for CAP, MI, stroke, and OF

The cost per index hospitalization was $13,825 for CAP, which was lower than all other conditions (Table 2). Because of the much higher incidence of CAP hospitalization compared to MI, OF, and stroke, the total cost of index CAP hospitalizations was much higher at $227.1 million compared to $205.2 million for MI, $81.9 million for stroke, and $127.8 million for OF. Similarly, total readmission costs associated with CAP were the highest at $23.3 million, compared to $11.6 million, $5.4 million, and $7.7 million for MI, stroke, and OF, respectively. Costs per readmission were similar between the conditions. The total costs, which include the sum of the index hospitalization costs and any associated readmission, totaled over $250.4 million for CAP compared to $216.8 million for MI, $87.3 million for stroke, and $135.6 million for OF (fig. 1).

During 2014, 7.6% of individuals received at least one pneumococcal vaccination and 45.31% received an influenza vaccination (Table 3). Health plan expenditures on pneumococcal and influenza vaccinations totaled $14.1 million ($7.3 PMPY) and $26.1 million ($13.4 PMPY), respectively. Member OOP costs made up < 1% of total expenditures as health insurance coverage typically includes the entire cost of preventive vaccinations. Preventive medications for MI and stroke were used by about 60% of the cohort in 2014 (Table 3). Combined costs of MI and/or stroke preventive medications exceeded $661 million ($339.33 PMPY). Prevention for OF was used by 6.3% of the cohort with total costs of $169 million ($86.74 PMPY).

**Table 3:** Preventive medication and vaccination use in 2014

Preventive measure	Individuals treated N (%)	Total health plan cost	Patient OOP	Total cost	PMPY^a^
Pneumococcal vaccination	147,552 (7.6%)	$14,142,934	$6930	$14,149,780	$7.26
Influenza vaccination	883,319 (45.3%)	$26,115,315	$14,643	$26,129,745	$13.40
Total	922,414 (47.3%)	$40,255,322	$21,573	$40,276,597	$20.66
MI/Stroke Prevention					
Antihypertensives	1,103,865 (56.6%)	$259,953,965	$120,376,142	$369,173,588	$189.38
Statins	754,659 (38.7%)	$146,574,868	$48,844,928	$194,778,686	$99.92
Aspirin	2514 (0.1%)	$11,569.57	8.40	11,573.70	0.01
Antiplatelets	108,899 (5.6%)	$49,170,046	$13,925,749	$63,053,238	$32.35
Anticoagulants	109,114 (5.6%)	$25,344,062	$9,197,641	$34,451,541	$17.67
Total MI/Stroke	1,225,741 (62.9%)	$481,054,511	$192,344,468	$661,468,627	$339.33
Bisphosphonates	98,129 (5.0%)	$26,495,337	$8,544,260	$34,128,920	$17.51
SERMs	11,437 (0.6%)	$12,752,847	$3,739,518	$16,482,842	$8.46
Denosumab	13,567 (0.7%)	$103,203,578	$3,602,221	$106,801,838	$54.79
Teriparatide	1261 (0.1%)	$10,494,134	$1,186,876	$11,680,624	$5.99
Total	121,870 (6.3%)	$152,945,896	$17,072,875	$169,094,223	$86.74

^a^Based on the Total Cost divided by the total population size

Abbreviations: *CAP*: community-acquired pneumonia; *MI*: myocardial infarction; *OF*: osteoporotic fracture; *ACEi*: angiotensin converting enzyme inhibitors; *ARBs*: angiotensin receptor blockers; *SERMs*: selective estrogen receptor modulators; *PMPY*: per person per year; *OOP*: out-of-pocket

## Discussion

MI, stroke, and OF are important disease states that contribute significant morbidity and mortality in individuals over 65 years of age. As such, they are a large focus of primary care providers and managed care organizations since there are effective preventive medications available. In this cohort representative of a MAPD insured population, the insurer spent over $661 million on medications for primary prevention of MI and stroke and an additional $169 million on primary prevention agents for OF. In contrast, only $14.1 million dollars were spent on pneumococcal vaccinations and $26.1 million on annual influenza vaccination – both effective preventive interventions for CAP [16].

In order to prevent CAP, the Infectious Diseases Society of America and the American Thoracic Society guidelines recommend smoking cessation, influenza vaccination, and pneumococcal vaccinations for adults aged ≥65 years and for those 19–64 years with chronic medical conditions [16]. Likewise, the current Advisory Committee on Immunization Practices guidelines recommend vaccination for adults aged ≥65 years with both PCV13 and PPSV23 vaccines in sequence [17]. Immunization will protect against influenza virus and vaccine serotypes of *Streptococcus pneumoniae* (Pneumococcus), which are among the most common causes and co-pathogens associated with pneumonia [17–21]. Pneumococcal vaccination decreases the rate of hospitalization associated with CAP and also lowers the risk of invasive disease such as pneumococcal meningitis and bacteremia [20–22]. Influenza vaccination has also been reported to reduce the risk of influenza-associated hospitalization for CAP [23].

The proportion of individuals receiving pneumococcal vaccination was 7.57% during 2015 in the cohort. Given that pneumococcal vaccination is generally once per lifetime in the elderly population, this can be considered a “yearly uptake” of vaccination in the population whereas the lifetime uptake is estimated to be nearly 60% [24, 25]. Similarly, less than 50% of the cohort received an influenza vaccination. This vaccination use falls short of the Healthy People 2020 goal of 90% pneumococcal and influenza vaccination in the older population [26]. Further, the uptake of the recommended two pneumococcal vaccine sequence was estimated to be only 18.3% in adults aged ≥65 years since the most recent guideline update highlighting that there are ample opportunities to improve adult pneumococcal vaccination rates [27].

Pneumococcal and influenza vaccination in adults over the age of 65 has been identified as a high-priority prevention service and vaccinations are recognized as ones of the most under-utilized but most effective, efficient, and cost-effective primary prevention services [28–31]. Even with 100% vaccination rate for influenza, the total costs would remain a fraction of the preventive costs for other diseases. Vaccinations are also annual or one-time interventions; thus, there are no recurring monthly costs or concerns for adherence compared to prevention for the other disease states. Several factors could contribute to low vaccination rates at the point of care including perceived financial and access issues, patient and physician knowledge of vaccination status or recommendations, as well as prioritization of care in the time window allotted for a wellness visit [32, 33]. Vaccinations may be prime candidates for increased intervention as it is relatively simple to implement by increasing awareness of vaccine safety, efficacy, and availability through managed care organizations or healthcare provider outreach to patient.

Access to vaccinations has increased due to provisions in the Affordable Care Act requiring zero cost-sharing [34] as well as the expanded role of community pharmacists to administer vaccinations [35, 36]. For seniors, Medicare coverage has been updated to allow compliance with the updated guidelines with coverage for the updated two-vaccination regimen [37]. Factors associated with vaccine uptake include perceptions about vaccine safety and whether a healthcare provider makes a recommendation [38–40]. Public awareness about the safety and efficacy of vaccinations must continue to be a priority for public health [41]. In addition, quality metrics by which health plans and physicians are measured should be updated to drive quality based on the most recent vaccination guidelines [42, 43].

The utilization and costs of vaccinations protecting against CAP display a clear disparity and an opportunity to provide more effective preventive efforts, especially when compared to the overall relative burden of CAP in this population. This is magnified when compared to MI, stroke, and OF and the yearly cost of preventive efforts spent for these disease states relative to the burden observed in the population. While it is warranted to focus on these more commonly feared diseases of the elderly, our study shows a tremendous burden of CAP hospitalizations in the older population that greatly exceeds that of MI, stroke, and OF – nearly double the hospitalization rates of the other disease states. Furthermore, total costs, LOS, readmissions, and associated mortality were comparable or higher with CAP-related hospitalizations than the other diseases.

### Limitations

Limitations common to studies using administrative claims data apply to this study [44–46]. These include a lack of certain information in the database (e.g. lab results, smoking status, weight, and health behavior information) and errors or omissions in claims coding. Data were from a single national MAPD health plan with a diverse population of older adults residing in broad geographic regions. Because this study uses data from a single insurer’s members only, the results may not be generalized to the general population. This study limited the cohort to members who did not have one or more hospitalizations in the 2014 pre-index period to focus on primary prevention costs rather than in a population with previous disease. This underestimates the burden of all of the disease states from a health plan perspective but does not bias the comparison between the disease states. The current study underestimated the burden of CAP since only inpatient CAP episodes, which have been shown to include only 40% of cases in the older population, were considered [1, 8]. Additionally, the study only considered primary hospital diagnosis. In cases of pneumonia, it is possible that hospitals code a different cause as the primary diagnosis if an underlying condition is exacerbated that requires serious management – thus further underestimating our estimated burden of CAP episodes. Medications or vaccinations obtained without utilizing an insurance benefit were unobserved in this study. This may include over-the-counter medications (e.g. aspirin to treat MI or stroke), cash payments for medications or vaccines, medications filled using low-cost generic programs [47], and vaccinations received through employers or other health fairs. This study also grouped together medication classes commonly used for primary prevention for MI and stroke. However, indications for each medication cannot necessarily be confirmed and may have been used to treat other conditions (e.g. anticoagulants prescribed for venous thromboembolism). Lastly, the data may have underestimated death as this was based on the Social Security Administration Death Master File for which some states ceased providing data in 2011. Also, discharge status of “expired” is not available given discharge date would represent the date of death which may jeopardize data de-identification.

## Conclusion

In this study of seniors with Medicare Advantage insurance coverage, the rate of CAP-related hospitalization was nearly double that of MI, stroke, and OF. CAP also had higher 30-day readmission rates than stroke and OF and increased mortality versus MI, stroke and OF. Despite higher incidence and high overall total costs, expenditures on preventive vaccinations were a fraction of the expenditures for these other disease states. Given suboptimal vaccination rates that fall below national goals, public health officials, healthcare providers, and managed care organizations should prioritize efforts to increase flu and pneumococcal vaccinations to reduce the significant burden of CAP in the older population.

## Appendix 1

**Table 4:** **Disease state ICD-9 Coding for outcomes and comorbidities.** ICD-9 codes used throughout the study

**Disease State**	**ICD-9 Codes**
Hospitalizations	
Community Acquired	1) 480.x-486.x, 487.0 Pneumonia as a principal diagnosis; OR
Pneumonia (CAP)	2) 518.8× Respiratory Failure or 038.xx Septicemia as a principal diagnosis with Pneumonia as a secondary diagnosis on the same claim line
Acute Myocardial Infarction	410.xx
Stroke	433.×1, 434.×1, 436.xx
Osteoporotic Fractures	
Hip, closed	820.0, 820.2, 820.8, 733.14
Forearm, closed	813.0, 813.2, 813.4, 813.8, 733.12
Pelvis, closed	808.0, 808.2, 808.4, 808.8
Distal femur, closed	821.0, 821.2, 733.15
Wrist, closed	813.4, 733.12
Humerus, closed	812.0, 812.2, 812.4, 733.11
Tibia/fibula, closed	823.0, 823.2, 823.8, 733.16
Clavicle, closed	810.0
	(excluding E-codes for fractures due to accident or trauma (*E800-E848, E881-E884, E908-E909, E916-E928*) and revision surgeries (ICD-9 procedure codes *00.71, 81.53, 78.6*; ICD-9 Diagnosis codes *733.81, 733.82*)
Comorbidities	
Chronic Pulmonary Disease	490.xx-496.xx, 500.xx-505.xx, 506.4
Cancer (Including Leukemia and Lymphoma)	140.xx-172.xx, 174.xx-195.xx, 200.xx-208.xx
Asthma	ICD-9 Diagnosis: 493.0×-493.2×
Heart failure	ICD-9 Diagnosis: 428.xx
Cardiomyopathy	ICD-9 Diagnosis: 425.xx
Diabetes	ICD-9 Diagnosis: 249.xx-250.xx, 648.xx
Liver disease	ICD-9 Diagnosis: 570.xx-573.xx, V10.017
Coronary artery disease	ICD-9 Diagnosis: 414.0×

## Appendix 2

**Table 5:** **Preventive medication coding algorithm.** Medication codes used throughout the study

**Medication class**	**GPI number (most specific level required)**
Medications to prevent pneumonia	
Influenza vaccine	1,710,002,020, 1,710,002,021, 1,710,002,023, 1,710,002,040, 1,710,002,044, 1,710,002,050, 1,710,002,061, 1,710,002,064 HCPCS G0008 CPT 90655, 90,656, 90,657, 90,658, 90,660, 90,662
Pneumococcal vaccine	1,720,006,500, 1,720,006,510, 1,720,006,530 HCPCS G0009, CPT 90669, 90,670, 90,732
Medications to prevent MI and/or stroke	
ACE Inhibitors	3610, 369,915, 369,918
ARBs	3615, 369,940
Beta-blockers	33, 369,920
Calcium channel blockers	34, 369,915
Diuretics	37, 369,918, 369,920, 369,940, 369,950, 369,955, 369,970, 369,980, 369,990
Anticoagulants (warfarin and NOACs)	8320, 83,370,030, 83,370,060, 83,337,010
Aspirin	64,100,010, 6,410,990,203, 6,410,990,204, 6,410,990,202, 6,410,990,210
Anti-platelet agents	8515
Statins	3940
Other cholesterol medications	39
Medications to prevent OF	
Bisphosphonates	300,420; HCPCS J3489
SERMs (raloxifene)	3,005,306,010
Denosumab	3,004,453,000; HCPCS J0897
Teriparatide	3,004,407,000; HCPCS J3110

## Appendix 3

**Table 6:** **Study cohort attrition.** Describes the cohort select process and total number of inclusions and exclusions

**Cohort Selection Step**	**Subjects remaining (N, %)**		**Subjects excluded (N,%)**	
1. All Medicare patients enrolled in Optum DOD between Jan 1, 2014 and Dec 31, 2015	3,971,785	100.000		
2. (1) and Medicare beneficiaries aged 65–89 years as of 1/1/2015	3,533,736	88.971	438,049	11.029
3. (2) and with continuous medical/pharmacy eligibility from 1/1/2014–12/31/2015 or death if occurs in 2015	1,993,580	50.194	1,540,156	38.777
4. (3) and excluding members with hospitalization for CAP, MI, stroke or OF during 2014	1951,510	49.134	42,070	1.059
5. (4) and excluding members with a cumulative stay ≥90 days in skilled nursing facility or long-term care during 2014	1,949,352	49.080	2158	0.054
**Final study sample**	**1,949,352**			

## Abbreviations

CAP: Community-acquired pneumonia
LOS: Length of stay
MAPD: Medicare Advantage with Prescription Drug Plan
MI: Myocardial infarction
OF: Osteoporotic fracture
